# Upper airway asymmetry in skeletal Class III malocclusions with mandibular deviation

**DOI:** 10.1038/s41598-017-12076-1

**Published:** 2017-09-22

**Authors:** De-Hua Zheng, Xu-Xia Wang, Dan Ma, Yuan Zhou, Jun Zhang

**Affiliations:** 1 Department of Orthodontics, School of Dentistry, Shandong University, Jinan, Shandong Province People’s Republic of China; 2 Department of Oral and Maxillofacial Surgery, School of Dentistry, Shandong University, Jinan, Shandong Province People’s Republic of China

## Abstract

The purpose of this study was to investigate the relationship between bilateral differences of upper airway and mandibular morphologic patterns in subjects with skeletal Class III mandibular deviation. 47 skeletal Class III (ANB < 0°) adult patients with and without mandibular deviation were divided into 2 groups. Bilateral differences of minimum cross-sectional area, mean cross-sectional area, volume of subdivisions (nasopharynx, palatopharynx, glossopharynx, hypopharynx) were assessed paired t test. Stepwise linear regression analysis and Pearson correlation coefficients were computed between a significant pair of upper airway variables and a pair of mandibular deviation variables to examine the quantitative relationship between the upper airway asymmetry and mandibular deviation. The mean cross-sectional area and the volume of palatopharynx on the deviated side in mandibular deviated group was significantly smaller than non-deviated side. The asymmetry index of the palatopharyngeal volume showed significant correlations with CRA asymmetry (r = 0.49) and Ramus asymmetry (r = 0.54). However, in the glossopharyngeal and hypopharyngeal segment, the mandibular deviated group showed significant asymmetry, characterized by larger mean cross-sectional area and volume in deviated side. The asymmetry index of the glossopharyngeal volume and hypopharyngeal volume showed significant correlations with CRA asymmetry (r = 0.42), Me-s (r = 0.72) and Me-s (r = 0.67) respectively.

## Introduction

Mandibular deviation is more frequently found in patients of skeletal Class III, which results from the excessive mandibular growth in the case of mandibular prognathism or a rotational and deviated position of the mandible. Considering the discrepancy in size or shape of the two halves or anatomical morphology of the mandible, subjects with mandibular deviation particular those with Class III malocclusion often present with differences in the hemi-mandibular volume, mandibular body length, ramal volume, mandibular body length, ramal volume, condylar length, condylar volume, and ramus inclination between the contralateral side of deviation and deviated sides^1–3^. Because of such asymmetric deformity, so called dental compensations, such as dental asymmetry, slanting of the occlusion plane, and unilateral crossbite, are commonly observed^4^. Moreover, imbalanced occlusion in patients with mandibular deviation can cause abnormal stress distribution on articular surfaces and dysfunctional osseous remodeling of condyles, causing the internal derangement and functional impairment of the temporomandibular joints (TMJs) and finally leading temporomandibular disorders (TMD)^5–7^. Furthermore, in patients with mandibular deviation, significant differences has been found in the volume of the medial pterygoid muscle^8^, electromyographic activity of masticatory muscle^9^ as well as the angle between the FH plane and the anterior border of the masseter muscle^10,11^. Therefore, subjects with mandibular deviation reportedly had asymmetric deformity of not only the hard tissue structures but also of the soft tissues when comparing the left and right sides.

The relationship between pharyngeal airway space and different craniofacial skeletal pattern morphology of patients, both anteroposterior (I, II, III skeletal class) and vertical (dolichofacial, mesofacial, brachyfacial) has been discussed in the orthodontic literature for many years, due to their proximity and intimate association^12–15^. Since many studies have demonstrated the airway constriction is the most dominating contributor to obstructive sleep apnea (OSA), much attention has been paid to ClassII patients which is characterized by narrower pharyngeal dimension and obstruction of the pharyngeal airway^16,17^. Several articles have demonstrated that patients with Class III malocclusion usually have the constriction of velopharynx and nasal cavity, nasal obstruction or choanal stenosis, which is caused by the severe maxillary hypoplasia^18–20^. For decades, lateral cephalometry has been used as a measurement method to examine airway size and shape, based on its high reproducibility and low radiation dose. However, the limitation of lateral cephalometric radiographs to evaluate the upper airway is to provide 2-dimensional (2D) images of complex 3-dimensional (3D) anatomic structures, which only shows anteroposterior measurements and fails to provide a full-scale view of the upper airway. More specifically, lateral cephalograms are not expect to offer reliable information on exact dimensions because of these limitations, such as magnification, distortion, superimposition of important structures, projection errors caused by vertical head rotation and difficulties in landmark identification^2,21^.

To overcome these limitations, cone-beam computed tomography (CBCT) has recently been brought into attention. The reason why cone-beam computed tomography (CBCT) is a reliable and reproducible method that can replace conventional lateral cephalograms is as follows^22^: (1) actual measurement without distortion regardless of head posture is possible; (2) real anatomic surface landmarks, not projected landmarks, are used for the dimensional measurements; (3) it enables volumetric measurements of hollow structures. (4) significantly reduced radiation dose compared with medical computed tomography machines and is equivalent to traditional dental imaging methods such as a full-mouth series. Moreover, specific software and their tools make it possible to obtain highly reliable measurements of osseous structures and facial characteristics, as well as to assess soft tissues in 3-dimensions, including measurements of the oropharynx volume, morphology, and minimum axial area. Many studies have been developed in this area^23–26^. Although various studies have been conducted for 3D volumetric depiction and morphological evaluation of the upper airway by using cone-beam computed tomography (CBCT), bilateral difference of upper airway in patients with mandibular deviation has not been previously described. Therefore, the purposes of the study were twofold: the first was to assess whether an asymmetry exists in subdivisions of upper airway (nasopharynx, palatopharynx, glossopharynx, orapharynx, hypopharynx) among patients with skeletal Class III mandibular deviation. The second purpose was to investigate the relationship between bilateral differences of upper airway and mandibular morphologic patterns in subjects with skeletal Class III mandibular deviation by evaluating cephalometric variables and volumes and cross-sectional areas of cone-beam computed tomography (CBCT) images of the upper airway.

## Methods

This research protocol was critically reviewed and approved by the Research Ethic Committee of Shandong University Dental School. The written informed consents were received from all parents, and the study was conducted according to the tenets of the Declaration of Helsinki for research involving human subjects. The methods were carried out in accordance with the approved guidelines of scientific reports.

Based on the mean standard deviation from a previous study^14^ and the formula of sample size calculation of group design described by Pandis^27^ (alpha value = 0.05, and the statistical power = 0.9), the simple size was finally decided of 47.

47 skeletal Class III (ANB < 0°) adult patients with and without mandibular deviation, who visited the Department of Orthodontics, School of Dentistry, Shandong University and Department of Stomatology for orthodontic treatment, participated in this study. 21 females (23 ± 2 years) and 26 males (24 ± 3 years) were retrospectively analyzed. The inclusion criteria were as follow: over 20 years of old; permanent dentition from the first permanent molar of one side to the other; no prior surgery for an injury involving the maxilla or the mandible, no disease syndromes; no allergic problems; no history of adenoidectomy; no pharyngeal pathology; no nasal obstruction and no obstructive sleep apnea. During CBCT scanning, patients were instructed to maintain an upright sitting posture and natural head position. The rest position of the tongue (in contact with anterior palate without touching the anterior teeth) and maximum intercuspation were also require. All of the scans were performed by the same researcher. Images were acquired using the CBCT scanner (KaVo Dental GmbH, Bismarcking, Germany) at a 0.30-voxel resolution with the scanning parameter of 120 Kv, 5 mA. The scan time was 8.9 seconds, and the slice thickness was 0.4 mm. The CBCT datasets were exported in the DICOM (Digital Imaging and Communications in Medicine) format.

Lateral cephalograms obtained from CBCT data were opened with the Dolphin Imaging program (version 11.0, Dolphin Imaging and Management Solutions, Chatsworth, Calif) to collect 4 angular (ANB, MM, FMA, SN-MP) measurements and 2 numeral (AF-BF, Wits) measurements. Tables 1 and 2 show definition of landmarks, reference planes, airway compartments and cephalometric measurements. All data were collected by an experienced operator. The patients’ self-reported height, weight, and BMI were extracted from the medical and dental history form, and organized by using Excel software (Microsoft, Redmond, Wash). According to clinical examination, patients were divided into 2 groups: 25 skeletal Class III patients without mandibular deviation (control group), 22 Class III patients with the occlusal plane inclined toward the ipsilateral side of the mandibular deviation (Deviated group). The clinical examination included: 1. Deviation of chin point: Deviation of the chin point was measured as the distance between the chin point and the facial midline directly on patients. The facial midline was defined as the perpendicular bisector of the line between the centers of the right and the left pupils. 2. Deviation of dental midlines: Deviation of dental midlines was defined as the horizontal distance between mesial contact points of maxillary central incisors and mandibular central incisors, measured directly on the patients. 3. Inclination of occlusal plane: Patients were asked to bite on a tongue blade, and then the cant in occlusal plane was detected with the angle between the blade and the inter-pupillary plane.

**Table 1:** Definition of Landmarks, Reference Planes and airway compartments.

	Landmarks/reference lines	Definition
Mandibular landmarks	CP	Most superior poin of coronoid process
	MdF	Most superior point of mandibular foramen
	MtF	Most superior point of mental foramen
	Me	Most inferior point on symphysis of mandible
	RCs	Consecutive points passing the lateral contour of mandibular ramus on a series of horizontal plane with 3 mm interval from mandibular angle to mandibular notch
	RL	Line approximating consecutive RCs projected onto coronal plane
	RL1	Reference line 7° to NSL
	RL2	Reference line perpendicular to NSL and intersecting the Sella point
	PSL	Line connecting SN and CP projected onto horizontal plane
Craniofacial landmarks	N	The most anterior point of the frontonasal suture in the mid-sagittal plane
	S	The central point of the pituitary fossa of the sphenoid bone
	A	The deepest anterior point in the concavity of the upper labial alveolar process
	B	The deepest anterior point in the concavity of the lower labial alveolar process
	PNS	The posterior point of the hard palate
	UT	The point of the uvula
	EB	The base point of epiglottis
	C_3_	The lowest point of the the third cervical vertebra
	Roof	The highest point of the airway in the mid-sagittal plane
	NSL	Line passing through the Sella and Nasion points
Cross-sectional planes	FH plane	An axial plane though orbitale point and porion point on both sides
	NR plane	The plane parallel to FH plane through Roof point
	PNS plane	The plane parallel to FH plane through PNS point
	UT plane	The plane parallel to FH plane through UT point
	EB plane	The plane parallel to FH plane through EB point
	C_3_ plane	The plane parallel to FH plane through C_3_ point
Pharyngeal airways	Nasopharynx (NP)	The pharyngeal airway above the PNS plane
	Oropharynx (OP)	The pharyngeal airway formed between the PNS and EB plane
	Palatopharynx (PP)	The pharyngeal airway formed between the PNS and UT plane
	Glossopharynx (GP)	The pharyngeal airway formed between the UT and EB plane
	Hypopharynx (HYP)	The pharyngeal airway formed between the EB and C_3_ plane

**Table 2:** Cephalometric Measurements.

	Measurement	Description
Anteroposterior skeletal pattern	ANB	Difference between SNA and SNB
	AF-BF (mm)	The distance between perpendiculars draw from A-point and B-point noto the Frankfort horizontal plane
	Wits (mm)	Distance from A-point and B-point parallel to the occlusal plane
Vertical skeletal pattern	MM	Angle formed by the maxillary (ANS-PNS) and the mandibular plane (Go-Me)
	FMA	Angle formed by the FH plane and the mandibular plane (Go-Me)
	SN-MP	Angle formed by the cranial base plane (SN) and the mandibular plane (Go-Me)
Mandibular morphology	CRA	Acute angle between the horizontal plane and RL
	HRA	Acute angle between the coronal plane and PSL
	Transverse ramus distance	Mean distance of RCs to midsagittal plane
	Ramus asymmetry	Difference between bilateral transverse ramus distances
	Me-S	Distance from Me to midsagittal plane
	MdF-S	Distance from MdF to midsagittal plane
	MtF-S	Distance from MtF to midsagittal plane
	Ramus-body Length	Distance from MdF to MtF

**Figure 1:**
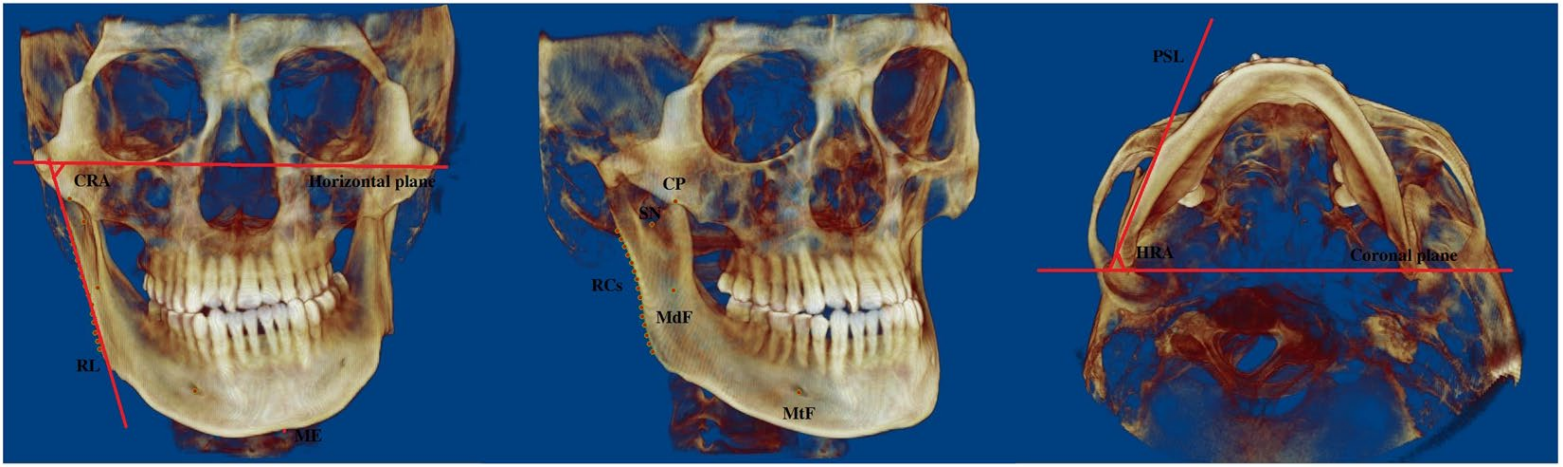
Landmarks and reference lines for 3D-CBCT evaluation. SN: sigmoid notch; CP: Coronoid process; MdF: Mandibular foramen; MtF: Mental foramen; RCs: Ramus contour point; Me: Menton; RL: Ramus line; CRA: Coronal ramus angle. PSL: Proximal segment line, the line connecting CP and SN projected no to horizontal plane; HRA: Horizontal ramus angle.

To evaluate upper airway asymmetry, a reference plane joining points sella turcica, nasion, and basion was selected as the midsagittal plane. The horizontal and coronal planes were perpendicular to the midsagittal plane with the horizontal plane passing through the bilateral midpoints between porion and orbitale and the coronal plane passing basion point. Landmarks and reference lines for 3D-CBCT evaluation (Fig. 1).

After identification of the PNS (posterior nasal spine), the superior border of the epiglottis, the point of uvula and C_3_ point (the third cervical vertebra) in the midsagittal plane, the upper airway was divided into three parts: the nasopharynx, oropharynx, and hypopharynx by the corresponding cross-sectional slices. The nasopharynx (NP) is the region from the top of the upper airway to posterior nasal spine, the oropharynx is located between posterior nasal spine and the superior border of the epiglottis and the hypopharynx (HYP) is defined as the region from the superior border of the epiglottis to the level of C_3_ point. The oropharynx was divided into 2 parts: the palatopharynx (PP, the hard palate plane to the point of the uvula) and the glossopharynx (GP, the point of the uvula to the superior border of the epiglottic). To evaluate upper airway asymmetry, the upper airway (NP, PP, GP, HYP) was divided into halves by midsagittal plane.

**Figure 2:**
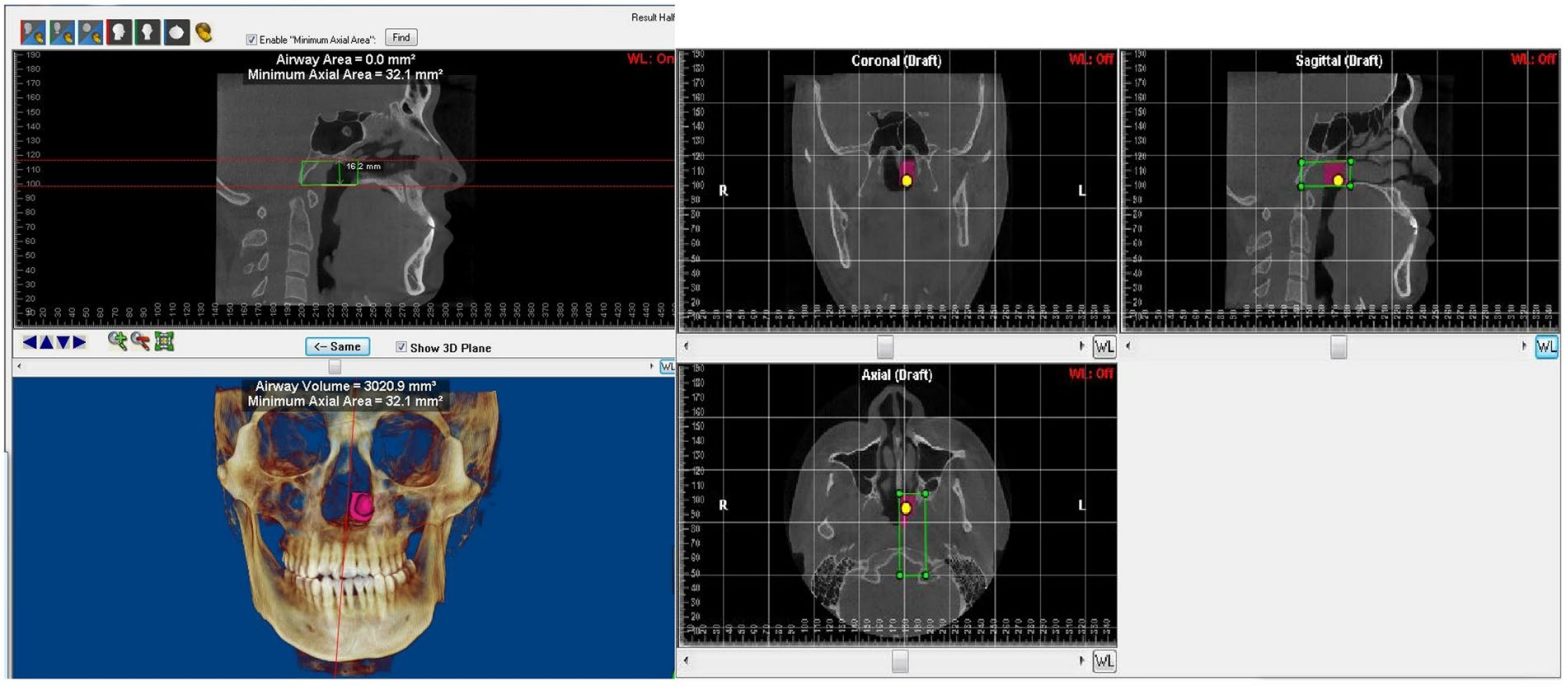
The region of mandibular deviated side of Nasopharynx (NP) and the 3D model of hemi-nasopharynx. Nasopharyngeal length, Mean cross-sectional area, Minimal cross-sectional area and volume.

**Figure 3:**
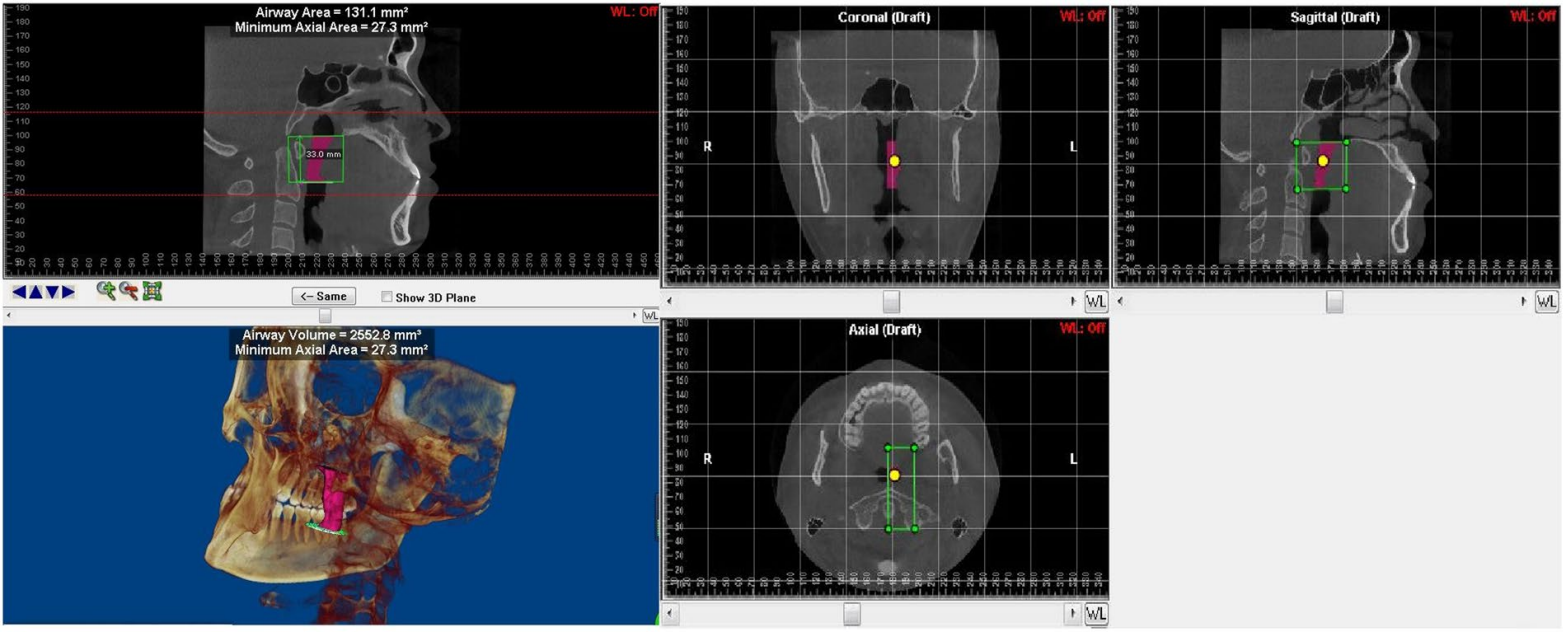
The region of mandibular deviated side of Palatopharynx (PP) and the 3D model of hemi- palatopharynx. Palatopharyngeal length, Mean cross-sectional area, Minimal cross-sectional area and volume.

**Figure 4:**
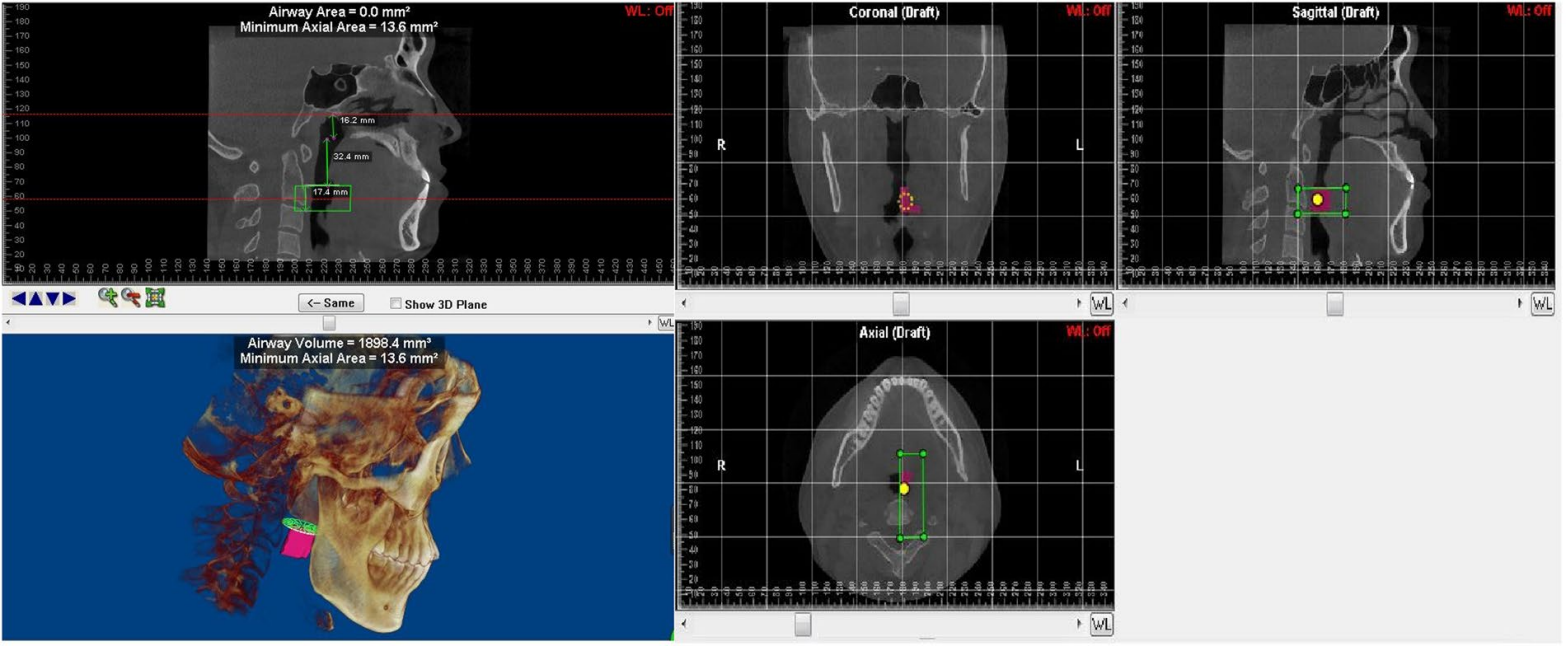
The region of mandibular deviated side of Glossopharynx (GP) and the 3D model of hemi- glossopharynx. Glossopharyngeal length, Mean cross-sectional area, Minimal cross-sectional area and volume.

**Figure 5:**
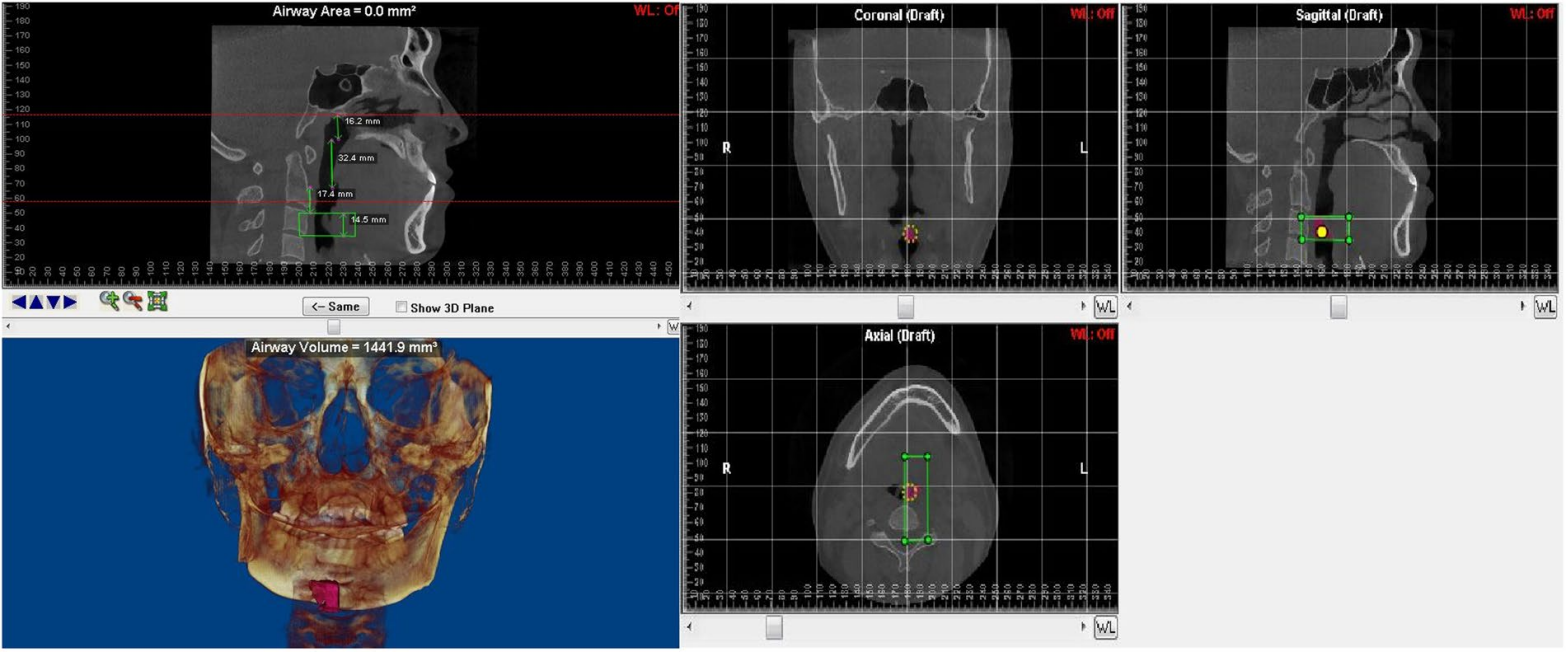
The region of mandibular deviated side of hypopharynx (HYP) and the 3D model of hemi- hypopharynx. hypopharyngeal length, Mean cross-sectional area, Minimal cross-sectional area and volume.

All measurements were made with Dolphin Imaging software. The volume, minimum cross-sectional areas and the height of each portion were measured with the tool for airway volume calculation in the 3-dimensional mode of software in the 50 (standard) threshold values. The limits for each portion of interest were defined in the cross-sectional slice and sagittal slice, the software automatically calculated the total volume and minimum cross-sectional area (CSA min) in the region previously set out. The mean cross-sectional area (CSAmean) of each region was computed as the ratio of Volume/segmental airway length. The upper airway divided into 4 parts: Nasopharynx (NP, Fig. 2) Palatopharynx (PP, Fig. 3) Glossopharynx (GP, Fig. 4) Hypopharynx (HYP, Fig. 5). The distances from each of the landmarks to the reference planes were measured by same observer. Bilateral difference in measurements indicated the asymmetry of the respective anatomic locus. Angulations of mandibular ramus were assessed in the coronal and cranio-caudal views. The mean distance of RCs to midsagittal plane was denoted as transverse ramus distance. The difference between bilateral transverse ramus distances was denoted as ramus asymmetry. The distance of menton to the midsagittal plane was denoted as menton deviation. The distance between mandibular and mental foramina was denoted as ramus-body length. Occlusal plane cant was assessed by difference between the distances of mesiobuccal cusps of bilateral maxillary first molars to horizontal plane.

**Figure 6:**
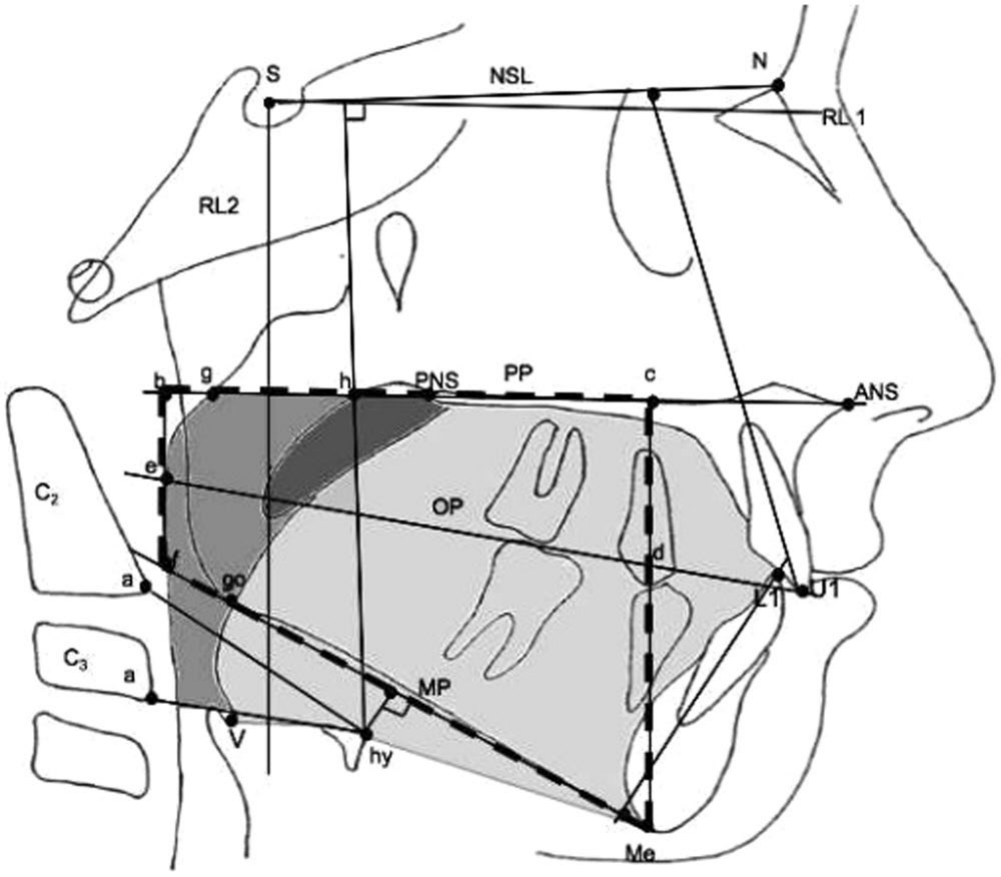
The cephalometric points, reference lines, and areas used in the study. Soft palate is black, tongue is light grey, and oropharyngeal area is dark grey.

The following landmarks were used to measure surrounding tissues of upper airway including: soft palate, hyoid, posterior pharyngeal wall, tongue. Hy, most anterior point on the hyoid bone; V (Vallecula), most profound point in the curvature of the depression just behind the root of the base of the tongue between the folds in the throat; a, most anteroinferior point on corpus of C2 and C3; g, point on the nasal surface of the soft palate at the level of maxillary plane (opposite point to h); h, point on the posterior pharyngeal wall at the same horizontal level as point g. Definitions of linear measurements: hy-NL, the perpendicular distance from NSL to hyoid; hy-MP, the perpendivular distance from MP to hyoid; hy-aC2, the linear distance between hy and aC2; hy-aC3, the linear distance between hy and aC3. Definitions of area measurements: black soft palate area bounded superiorly by PP; oropharyngeal area, the dark grey oropharyngeal area, bounded superiorly by a backward extension of the maxillary plane drawn through the tip of epiglottis; tongue area: light grey area enclosed posteriorly by the oropharynx and uvula, superiorly by the hard palate, and anterior by the lingual aspects of the anterior teeth and lingual mandibular symphyseal contour. The inferior border is the line extending from the vallecular to the most anterior point on the hyoid body and the line from the most anterior point on the hyoid bone to the menton (Fig. 6).

### Statistical analysis

Independent sample *t* test were used to compare the anteroposterior and vertical position of the maxilla and the mandible between Class III mandibular deviated group and control group. Independent sample *t* test were also used to compare asymmetric index of upper airway as well as measurements of surrounding tissue between Class III mandibular deviated group and control group. Statistical values, including mean and SD, were computed for mandibular morphologic measurements and upper airway discrepancy on the mandibular deviated and no-deviated sides in each group. Subsequently, the asymmetry index was computed by subtracting the values on the mandibular deviated side from those of the no-deviated side for each measurement, and the statistical values of this asymmetry index were also computed in each group. Statistical differences of each measurement between deviated side and non-deviated side in mandibular deviation group were examined with a paired *t* test.

Stepwise linear regression analysis was completed to obtain a significantly valid mandibular deviation for describing upper airway asymmetry using upper airway variables and mandibular deviation variables as response variables and explanatory variables (F value > 5.0). Then Pearson correlation coefficients were computed between a significant pair of upper airway variables and a pair of mandibular deviation variables to examine the quantitative e relationship between the upper airway asymmetry and mandibular deviation.

Twenty randomly selected CT images were remeasured by the same investigator after one month to assess intra-rater reliability. Pair *t* tests were used to estimate systemic errors, and it was determined that all measurements were free of systemic errors. The random error was estimated with formula^28^: ME^2^ = ∑d^2^/2n (d is deviation between the two measurements; n is the number of paired double measurements). The random errors varied from 0.23 to 0.34 mm in 3D linear measurement, from 12.15 to 26.24 mm^2^ in area measurements, and from 16.75 to 20.45 mm^3^ in volume measurements. The interinvestigator differences for cephalometric measurements were evaluated with paired *t* test at *P* < 0.05, and there were no significant differences.

## Result

**Figure 7:**
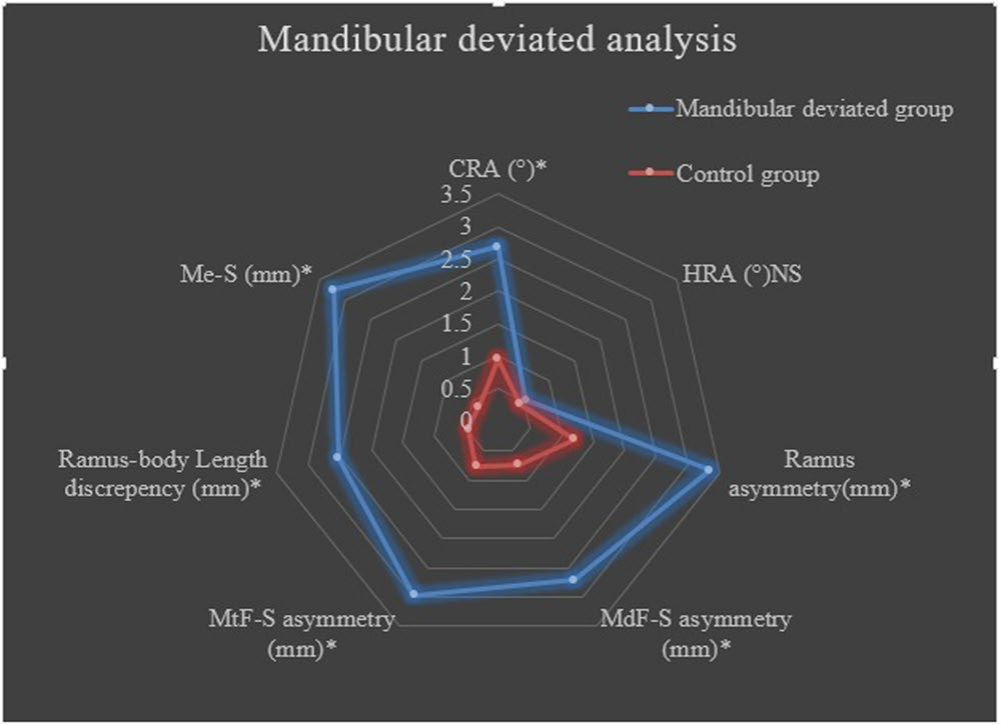
Mandibular deviated analysis. The discrepancy of asymmetry index between mandibular deviated group and control group. *P < 0.05 NS, not significant.

The groups consisted of 22 Class III mandibular deviated adult patients (11 females, 11 males), 25 Class III without mandibular deviation (control group, 11 females, 14 males). Demographic characteristics of participants in two groups was shown in Table 3. The cephalometric anteroposterior and vertical position of the maxilla and mandible of Class III mandibular deviated group and control group are compared in Table 4. There were no statistically significant differences between the Class III mandibular deviated group and control group. Comparison of asymmetry index of mandibular measurements was showed in Table 5 and Fig. 7.

**Table 3:** Demographic characteristics of participants in two groups.

Variable	Deviated group N = 22	Non-deviated group N = 25	*p*-Value
**Gender**			
Male	12	14	*P* > 0.05
Female	10	11	*P* > 0.05
**Age**			
20–24	11	12	*P* > 0.05
24–27	11	13	*P* > 0.05

*p*-values calculation was done using chi-square test.

**Table 4:** Descriptive statistics of cephalometric measurements of patients in two groups, classified according to mandibular deviation.

	Class III deviated group (n = 22)	Class III control group (n = 25)	*P*
	Mean (SD)	Mean (SD)	
**Anteroposterior skeletal pattern**			
ANB (°)	−1.92 (2.62)	−2.43 (2.74)	>0.05
AF-BF (mm)	0.15 (3.40)	0.23 (3.25)	>0.05
Wits (mm)	4.78 (1.72)	5.21 (1.21)	>0.05
**Vertical skeletal pattern**			
MM (°)	27.82 (4.62)	32.56 (4.32)	>0.05
FMA (°)	24.21 (4.72)	27.31 (4.91)	>0.05
SN-MP (°)	33.21 (4.82)	35.11 (5.87)	>0.05

P < 0.05.

**Table 5:** Comparison of asymmetry index of mandibular measurement (Mean, SD).

Variables	Mandibular deviated group (n = 22)	Control group (n = 25)	*P*
CRA (°)	2.69 ± 0.52	0.95 ± 0.37	0.000
HRA (°)	0.56 ± 0.35	0.44 ± 0.57	0.000
Ramus asymmetry (mm)	3.34 ± 1.12	1.20 ± 0.65	0.000
Me-S (mm)	3.26 ± 0.35	0.38 ± 0.29	0.000
MtF-S (mm)	2.71 ± 1.02	0.73 ± 0.28	0.000
MdF-S (mm)	2.97 ± 0.87	0.76 ± 0.32	0.000
Ramus-body Length (mm)	2.52 ± 1.14	0.47 ± 0.54	0.000

P < 0.001.

With regard to surrounding tissues of upper airway comprising soft palate, hyoid, posterior pharyngeal wall and tongue, no significant difference was found between the Class III mandibular deviated group and control group (Table 6). Comparison of cross-sectional area and volume of upper airway between the deviated group and control group was shown in Table 7.

**Table 6:** Comparison of surrounding tissue measurements of upper airway (Mean, SD).

Variables	Mandibular deviated group (n = 22)	Control group (n = 25)	*P*
Soft palate area (mm^2^)	3.41 ± 0.72	3.29 ± 0.86	>0.05
Tongue area (mm^2^)	37.05 ± 4.95	35.82 ± 5.02	>0.05
Oropharyngeal area (mm^2^)	7.52 ± 2.20	6.57 ± 1.93	>0.05
hy-MP (mm)	2.31 ± 1.27	2.46 ± 1.35	>0.05
hy-NL (mm)	12.82 ± 2.13	13.08 ± 2.27	>0.05
hy-aC_2_ (mm)	5.37 ± 0.92	5.47 ± 1.12	>0.05
Hy-aC_3_ (mm)	4.65 ± 1.31	5.03 ± 0.94	>0.05

P < 0.05.

**Table 7:** Comparison of cross-sectional area and volume of upper airway between the deviated group and control group (Mean, SD).

	Class III deviated group (n = 22)	Class III control group (n = 25)	
**Nasopharynx (NP)**			
CSAmean (mm^2^)	427.91 ± 79.89	401.76 ± 80.82	>0.05
NPvolume (mm^3^)	8056.21 ± 1854.87	8237.28 ± 1948.71	>0.05
**Palatopharynx (PP)**			
CSAmean (mm^2^)	388.26 ± 156.32	402.45 ± 178.87	>0.05
PPvolume (mm^3^)	9783.12 ± 3029.23	9731.21 ± 2775.53	>0.05
**Glossopharynx (GP)**			
CSAmean (mm^2^)	336.60 ± 114.41	386.34 ± 223.52	0.000
GPvolume (mm^3^)	5068.92 ± 2232.54	6653.53 ± 2959.47	0.000
**Hypopharynx (HYP)**			
CSAmean (mm^2^)	283.78 ± 112.80	357.22 ± 158.62	0.000
HYPvolume (mm^3^)	4774.50 ± 1979.16	5211.52 ± 2321.45	0.000

P < 0.05.

### Characteristic upper airway asymmetry in skeletal Class III patients with mandibular deviation

In nasopharyngeal airway, no statistically significant differences were observed in any assessed measurements in bilateral sides both in mandibular deviated group and control group (Tables 8 and 9).

**Table 8:** Comparison of asymmetry index of upper airway. (Mean, SD).

	Class III deviated group (n = 22)	Class III control group (n = 25)	P
Variables	Asymmetry index	Asymmetry index	*P*
**Nasopharynx (NP)**			
CSAmean (mm^2^)	30.97 ± 23.99	29.83 ± 22.45	>0.05
NPvolume (mm^3^)	518.79 ± 373.86	498.65 ± 383.42	>0.05
**Palatopharynx (PP)**			
CSAmin (mm^2^)	5.34 ± 4.14	5.12 ± 4.28	>0.05
CSAmean (mm^2^)	20.65 ± 29.25	16.34 ± 17.79	0.000
PPvolume (mm^3^)	337.10 ± 287.56	186.93 ± 173.84	0.000
**Glossopharynx (GP)**			
CSAmin (mm^2^)	4.27 ± 4.17	4.14 ± 4.02	>0.05
CSAmean (mm^2^)	19.77 ± 14.44	11.17 ± 10.43	0.000
GPvolume (mm^3^)	398.64 ± 338.92	87.34 ± 109.47	0.000
**Hypopharynx (HYP)**			
CSAmean (mm^2^)	23.78 ± 16.80	17.22 ± 13.58	0.000
HYPvolume (mm^3^)	381.50 ± 279.16	221.49 ± 119.32	0.000

P < 0.05.

**Table 9:** Mean measurements of the upper airway (Mean, SD).

Variable	Class III deviated group (n = 22)		*P*	Class III control group (n = 25)		*P*
	Deviated side	Non-deviated side		Left side	Right side	
**Nasopharynx (NP)**						
CSAmean (mm^2^)	214.78 ± 81.46	222.82 ± 89.2	>0.05	218.2 ± 91.34	229.87 ± 86.47	>0.05
NPvolume (mm^3^)	3891.10 ± 1898.99	4129.90 ± 1947.98	>0.05	4023.12 ± 1702.1	4403.53 ± 1412.74	>0.05
**Palatopharynx (PP)**						
CSAmin (mm^2^)	33.03 ± 29.78	36.88 ± 33.84	0.021	36.42 ± 21.83	37.63 ± 30.12	0.032
CSAmean (mm^2^)	183.42 ± 109.77	204.06 ± 126.06	0.003	194.32 ± 120.36	202.93 ± 132.86	>0.05
PPvolume (mm^3^)	4546.50 ± 2700.57	4883.60 ± 2776.21	0.000	4591.23 ± 2857.23	4692.32 ± 2736.76	>0.05
**Glossopharynx (GP)**						
CSAmin (mm^2^)	36.30 ± 36.33	34.72 ± 37.84	>0.05	33.47 ± 28.65	32.67 ± 30.84	>0.05
CSAmean (mm^2^)	181.13 ± 100.37	161.35 ± 93.79	0.000	185.43 ± 111.43	179.32 ± 100.86	>0.05
GPvolume (mm^3^)	3497.50 ± 2165.66	3098.86 ± 1943.10	0.000	3283.12 ± 1963.32	3296.43 ± 1875.23	>0.05
**Hypopharynx (HYP)**						
CSAmean (mm^2^)	163.52 ± 76.20	139.75 ± 63.08	0.000	179.56 ± 69.34	162.84 ± 61.07	>0.05
HYPvolume (mm^3^)	2684.66 ± 1375.75	2303.17 ± 1161.26	0.000	2756.43 ± 1406.59	2543.92 ± 1294.72	>0.05

P < 0.05.

The mean cross-sectional area (CSAmean), the minimum cross-sectional area (CSAmin) and the volume of palatopharynx on the deviated side in mandibular deviated group was significantly smaller than non-deviated side. The asymmetry index of palatopharyngeal mean cross-sectional area (CSAmean) and volume in mandibular deviated group was signigicantly larger than in the control group. No significant asymmetry of minimum cross-sectional area (CSAmin) was found between mandibular deviated group and control group.

In the glossopharyngeal segment, the Class III mandibular deviated group showed significant asymmetry, characterized by larger mean cross-sectional area and volume in deviated side. However, no statistically asymmetry was observed in glossopharyngeal minimum airway area in both Class III mandibular deviated group and control group.

In the hypopharyngeal portion, the mean cross-sectional area and volume on the deviated side in mandibular deviated group was significantly larger than non-deviated side. The mean value of the asymmetry index in the mandibular deviated group was significantly larger than control group.

### Relationship between mandibular deviation and upper airway asymmetry

Stepwise linear regression analysis and significant correlation coefficients between upper airway volume and mandibular measurements were showed in Table 10.

**Table 10:** Stepwise linear regression analysis and significant correlation coefficients between upper airway volume and mandibular measurements.

Response variable (upper airway volume)	Explanatory variable (mandibular measurements)	*F* value	Correlation coefficients
**Palatopharyngeal volume**			
Deviated side	CRA asymmetry	9.21	−0.41*
	Ramus asymmetry	14.35	−0.51**
Non-deviated side	CRA asymmetry	8.31	0.40*
	Ramus asymmetry	15.28	0.52**
Asymmetry index	CRA asymmetry	12.54	0.49*
	Ramus asymmetry	16.24	0.54**
**Glossopharyngeal volume**			
Deviated side	CRA asymmetry	7.36	0.39*
	Me-s	21.67	0.60**
Non-deviated side	CRA asymmetry	6.74	−0.38*
	Me-s	19.38	−0.57**
Asymmetry index	CRA asymmetry	10.24	0.42*
	Me-s	34.71	0.72***
**Hypopharyngeal volume**			
Deviated side	Me-s	16.55	0.53**
Non-deviated side	Me-s	15.10	−0.52**
Asymmetry index	Me-s	29.49	0.67***

*P < 0.05; **P < 0.01; ***P < 0.0001.

Ramus asymmetry coupled with CRA asymmetry was found to be valid parameter for palatopharyngeal volume on both the mandibular deviated side, non-deviated side and the asymmetry index of palatopharyngeal volume Table (10). Significant correlations were found between Ramus asymmetry and palatopharyngeal volume (deviated side, r = −0.51; non-deviated side, r = 0.52). A significant correlation was also found between CRA asymmetry and palatopharyngeal volume on the mandibular deviated side (r = −0.41) and the mandibular non-deviated side (r = 0.40). Similarly, the asymmetry index of the palatopharyngeal volume showed significant correlations with CRA asymmetry (r = 0.49) and Ramus asymmetry (r = 0.54).

CRA asymmetry coupled with menton deviation (Me-s) was found to be valid parameter for glossopharyngeal volume on both the mandibular deviated side, non-deviated side and the asymmetry index of glossopharyngeal volume. Significant correlations were found between Me-s and glossopharyngeal volume (deviated side, r = 0.60; non-deviated side, r = −0.57). A significant correlation was also found between CRA asymmetry and glossopharyngeal volume on the mandibular deviated side (r = 0.39) and the mandibular non-deviated side (r = −0.38). Similarly, the asymmetry index of the glossopharyngeal volume showed significant correlations with CRA asymmetry (r = 0.42) and Me-s (r = 0.72).

Menton deviation (Me-s) was found to be a valid parameter for hypopharyngeal volume on both the mandibular deviated and non-deviated sides and the asymmetry index of hypopharyngeal volume. A significant correlation was found between chin deviation (Me-s) and hypopharyngeal volume on the mandibular deviated side (r = 0.53) and non-deviated side (r = −0.52). Similarly, the asymmetry index of hypopharyngeal volume showed a significant correlation with Me-s (r = 0.67).

## Discussion

Abnormal morphology of the upper airway can make the airway narrower and prone to breathing disturbances. More importantly, respiratory dysfunction could cause increased morbidity and mortality in a condition like obstructive sleep apnea (OSA) which is greatly relevant to orthodontic diagnosis and treatment planning^29,30^. Because a close relationship between craniofacial morphology features and upper airway dimension in patients with malocclusion, previous researches emphasized much on the impacts of anteroposterior position of mandible and vertical skeletal pattern on volumes and cross-sectional areas of upper airway. When the Angle skeletal classification is taken into account, it was observed that Class І and Class III subjects had significant larger airway volumes compared with Class II subjects. Many studies^31,32^ have reported that the retro position of the mandible and increased upper or lower face heights were primary reasons for airway narrowing in patients with skeletal Class II malocclusion. On the other hand, estimates about the dimension of upper airway in patients with skeletal Class III malocclusion remain controversial. Grauer *et al*.^33^ reported that the volume of the pharyngeal airway did not differ significantly between the Class III and Class І groups. However, several studies^34,35^ reported that the volume of the pharyngeal airway was significantly greater in the Class III group than in the Class І groups. Although the relationship between pharyngeal characteristics and different dentofacial skeletal patterns has been intensively researched, the pharyngeal morphology of patients with mandibular deviation remain ambiguous. Considering the close relationship between pharynx structures and craniofacial complex growth and development, mandibular deviation, which is a frequent manifestation in Class III patients with mandibular prognathism could have impact on pharyngeal morphology. In other words, since morphological dimension of upper airway is complex due to the geometric interplay of dentition, bone, and soft tissues, an association could be expected to exist between mandibular deviation and abnormal morphology of upper airway. This is the first study to assess the correlation between asymmetric pharyngeal dimensions and mandibular deviation. Based on our main findings, in addition to nasopharynx, asymmetric dimensions of oropharynx and hypopharynx were observed in skeletal Class III patients with mandibular deviation.

To explore the relationship between upper airway asymmetry and mandibular deviation, reliable assessment of morphology in each part of deviated mandibular is the primary problem. According to the analysis of Park *et al*.^36^, the mandible has six distinct functional units, and dentofacial deformity with malocclusion can be interpreted as their unbalanced growth. Additionally, the mandibular and mental foramina was important reference point located at the junction of the skeletal units and landmark point where primary intramembranous ossification starts, respectively. As for mandibular deviation, it may occur due to right and left condylar or ramal vertical dimensional discrepancies, differences between the corpus lengths of the 2 sides or deviated position of the chin. Hence, in our study, angular and linear measurements to represent the condylar, coronoid, ramus, body and chin units were used. We measured transverse distance of unilateral ramus (MdF-S), transverse distance of unilateral body (MtF-S) and Ramus-body length on the basis of the mental foramen and mandibular foramen. Moreover, the asymmetry index of coronal ramus angle and horizontal ramus angle were used to measure the angular discrepancy of deviated mandibular rami. Furthermore, the distance of menton to midsagittal plane was denoted as menton deviation which represent the position of the chin unit.

There were many potential influences on airway dimensions and shape. This study controlled for the following factors:

1. Airway differences related to patients age. It has been demonstrated that airway growth ceases between the ages of 18 and 20 years^37,38^. Goncalves *et al*.^39^ asserted that the growth pattern of the upper airway width exhibits a plateau from 6 to 9 years, a linear increase from 9 to 16 years, and another plateau from 16 to 18 years. Schendel *et al*.^40^ observed similar results with an airway increase until the age of 20 years, when a variable periods of stability occurs, and a slow decrease after the age of 40. Therefore, subjects enrolled in this study are over 20 years old, so as to ensure airway volume and shape did not correlate with age.
2. The influence of anteroposterior and vertical skeletal pattern on airway morphology. Although ANB angle is the most used criteria in the determination of the anteroposterior relationship between the maxilla and the mandible, it might be influenced by the anteroposterior position of nasion relative to Points A and B, and some authors have suggested that the diagnosis of such discrepancies should be based on more than 1 anteroposterior appraisal^41–44^. Therefore, our sample included subjects with skeletal Class III malocclusion according to ANB angle, AF-BF and wits appraisal. No significant differences of these measurements were found in both mandibular deviated group and control group. Similarly, MM^45^, FMA^46^ and SN-MP^47^ showing the vertical skeletal pattern, were no significant differences between mandibular deviated group and control group. According to vertical measurements, no subject with severe mandibular hypodivergency or hyperdivergency was included in the sample, because this aspect can influence airway dimensions, as described by Joseph *et al*.^48^. Based on two aspects above, it is unlikely that anteroposterior and vertical position of the mandible contribute to the differences that we noted in airway morphology.

In our study, significant asymmetry was found in oropharyngeal and hypopharyngeal. In the palatopharyngeal segment, the volume and mean cross-sectional area were significant larger on the non-deviated side than on the deviated side. Moreover, the asymmetry index of the volume and mean cross-sectional area of palatopharyngeal was significantly correlated with deviated mandibular ramus (CRA, Ramus asymmetry). To explain these results, we suggested that mandibular deviation could cause abnormalities of the bony cage enveloping the oropharyngeal and hypopharyngeal cavity. As for palatopharyngeal, deviated mandibular rami may contribute to the decrease of the volume and cross-sectional area of deviated side by imbalanced muscular force and laterally displacement of soft tissues surrounding palatopharyngeal. Significant high correlation coefficients between asymmetry index of palatopharynx and deviated mandibular rami suggested that the greater mandibular rami deviation, the greater asymmetric volume and cross-sectional area between the mandibular deviated side and non-deviated side. Unlike the palatopharyngeal segment, the volume and mean cross-sectional area were significant larger on the mandibular deviated side than on the non-deviated side in glossopharynx and hypopharynx. Stepwise linear regression analysis selected only menton deviation (Me-S) as a valid parameter for the asymmetry index of hypopharyngeal volume and cross-sectional area. Several previous studies mentioned that Menton deviation correlates with several skeletal abnormalities of mandible, including elongated mandibular body, discrepant hemi-ramal and condylar volume, and asymmetric ramal inclination between the non-deviated and deviated sides^49,50^. It is one of the most prominent features in skeletal Class III patients with mandibular deviation which usually determines the degree of facial asymmetry^51^. This suggested that hypopharyngeal asymmetry could be attributed to menton deviation in mandibular deviation group. Since the position of the tongue, lateral pharyngeal walls, muscular attachment and mandible are closely related, asymmetric glossopharynx and hypopharynx may result in displacement of the above structure. On the other hand, menton deviation may result in laterally positioned attachment of genioglossus and geniohyoideus, which were most glossopharynx and hypopharynx-related muscles. This is probably one of the reason why asymmetric volume and cross-sectional area exist in these two portions.

In this study, CBCT was the selected diagnostic method to evaluate the asymmetric morphology of the upper airway. Although CBCT does not show clear delineations between soft tissues, it clearly demonstrates the airway space and related skeletal structures and has been shown to provide precise and clinically relevant information on upper airway dimensions. Furthermore, CBCT which is widely used in dentistry, has lower associated scanning costs than magnetic resonance imaging (MRI) and can be used for orthodontic diagnosis and treatment planning. Therefore, MRI is rarely necessary to study orthodontic patients without any history of pharyngeal disease. By using Dolphin Imaging program, researchers can reconstruct 3D models and visualize various craniofacial structures of interest. These are the reasons why we choose CBCT records to carry out our study. However, when compared with magnetic resonance imaging (MRI), our study does exist limitations. The upper airway is enclosed along its length by bones, including the nasal turbinate, hard palate of the maxilla, mandible, hyoid, and by soft tissues, including tongue, soft palate, tonsillar pillars, pharyngeal fat pads. A big challenge to distinguish pharyngeal fat pads between lateral pharyngeal wall and mandibular ramus is that CBCT records is difficult to reliably delineate borders among soft tissues. Therefore, whether the lateral pharyngeal wall and pharyngeal fat pads are related with upper airway asymmetry is still unclear.

When the surrounding soft tissues of upper airway are taken into account, soft palate and tongue are of particular importance in analyzing the contributing factor of asymmetric upper airway. Therefore, the assessment of soft palate and tongue, represented by soft palate area, tongue area and hyoid position was considered necessary. According to our study, no significant differences were observed in above aspects between mandibular deviated group and control group. Considering our results, the soft palate and tongue might not the contributing factors of asymmetric upper airway in mandibular deviated group. Although the tonsillar tissues and adenoidal structures were considered in our study, we could barely detect significant structures of tonsil and adenoid in our subjects. The reasons may be as follows: on the one hand, assessment of adenoid is difficult, especially in adult without a history or clinical evidence of nasopharyngeal disease, because by 10 years of age, the adenoids begin to regress and then gradually diminish in size throughout adulthood^52^. On the other hand, it is difficult to distinguish tonsillar structures from surrounding soft tissues of pharynx because of low discrimination of soft tissues.

The extent to which various pathogenic factors contribute to the phenomenon of obstructive apneas and hypopneas probably varies from patient to patient. In general, upper airway collapsibility is a function of the balance of surrounding tissue collapsing pressure, intraluminal pressure, and compliance of pharyngeal walls. Pharyngeal compliance is expressed as the change in volume or cross-sectional area per unit change in pressure and is an indicator of the ease with which an airway can be deformed. Although asymmetric morphology of upper airway may contribute to upper airway collapsibility and pharyngeal compliance, data are insufficient to clarify its role in the pathogenesis of obstructive apneas and hypopneas. Therefore, exploring the relationship between abnormities of pharyngeal lumen and obstructive apneas might be meaningful. Orthognathic surgery combined with orthodontics is often required to correct severe skeletal discrepancies in skeletal Class IIIpatients with mandibular deviation^53^. However, mandibular setback surgery coupled with correction of deviated mandible can cause a decrease in the airway space, and this decrease in airway size might cause obstructive apneas and hypopneas^54–56^. Whether the asymmetric morphology of upper airway in skeletal Class III patients with mandibular deviation has its role in postsurgical decreased pharyngeal dimensions is still uncertain. Therefore, an understanding of airway morphology should be highlighted especially in those patients who are subject to orthognathic surgery.

## Conclusions

1. Apart from nasopharyngeal segment, significant asymmetry was found in other parts of upper airway (palatopharynx, glossopharynx, hypopharynx) both in volume and mean cross-sectional area among patients with skeletal Class III mandibular deviation.
2. The volume and mean cross-sectional area of glossopharynx and hypopharynx in patients with skeletal Class III mandibular deviation were significantly larger on the mandibular deviated side and smaller on the non-deviated side. In contrast, significantly smaller volume and mean cross-sectional area of palatopharynx was found on the mandibular deviated side.
3. The asymmetry of the palatopharynx and hypopharynx is statistically related to deviated mandibular ramus (CRA, Ramus asymmetry) and deviated menton (Me-S) respectively. However, the asymmetry of glossopharynx is statistically related to not only mandibular ramus (CRA asymmetry) but also deviated menton (Me-S).
